# A longitudinal study of the association between visual impairment and income change using a national health screening cohort

**DOI:** 10.1038/s41598-022-05003-6

**Published:** 2022-01-19

**Authors:** Hyo Geun Choi, Min Joung Lee, Sung Uk Baek

**Affiliations:** 1 Hallym Data Science Laboratory, Hallym University College of Medicine, Anyang, South Korea; 2 Department of Otorhinolaryngology-Head & Neck Surgery, Hallym University College of Medicine, Hallym University Sacred Heart Hospital, Anyang, South Korea; 3 Department of Ophthalmology, Hallym University College of Medicine, Hallym University Sacred Heart Hospital, 22, Gwanpyeong-ro 170beon-gil, Dongan-gu, Anyang-si, Gyeonggi-do 14068 South Korea

**Keywords:** Environmental social sciences, Diseases, Health care

## Abstract

We evaluated the influence of visual impairment (VI) on income change using the longitudinal database of a Korean National Health Insurance Service cohort. A total of 5292 participants ≥ 40 years old and registered as visually impaired persons were selected at a 1:4 ratio with 45,081 non-VI participants matched for age, sex, and income level. The income level of both the VI and non-VI groups increased over time. In the VI group, the income levels 3, 4 and 5 years were higher than the initial value, while the income levels from 1 through 5 years were increased each year in the non-VI group. The rate of change in income between time and VI were significant. In the subgroup analysis considering age, sex, and severity of VI, the rate of change in income were significant in < 65 years old subgroups. Regarding the severity of VI, a significant interaction was found for the mild-to-moderate VI subgroup. Although both the VI and non-VI groups showed increased income levels over 5 years, the degree of income increase in the VI group was relatively lower than that in the non-VI group. This finding was prominent in the middle-age subgroup. These results strongly suggested that VI induced an income inequality.

## Introduction

Visual impairment (VI), including blindness, is a serious disability that has a strong impact on quality of life. In particular, VI is well-known to be associated with lower socioeconomic status (SES)^1–6^. A relationship between VI and income level has been reported in diverse studies^2–5^; the results, varying between non-significant and significant associations, implicate a complex link between the two factors.

It is frequently asserted that VI is both a consequence and a cause of low income^3,5–7^. Whereas some evidence suggests that low income is a major barrier to uptake of ophthalmologic care and leads to lower medical compliance^8–10^, other evidence indicates that VI reduces the earning potential of impaired patients and/or the household members who care for them^7,11^.

Due to the multifactorial nature of social-economic status, identification of factors affecting income level is non-trivial, and certainly, there are numerous confounding factors that need to be considered and controlled for^1,3,12^. In this respect, the majority of previous studies’ cross-sectional design is a limiting factor, as it does not allow for analysis of any temporal relationship between visual impairment and income change^1,4,13–16^. In order to clarify the association between these two factors, a longitudinal study design with a large population-based database is required.

A comprehensive understanding of the link between VI and income can be utilized to make socioeconomic policy that effectively supports people with VI. Furthermore, it might help to break the social and personal links between those two factors. The current study’s purpose was to evaluate VI’s impact on longitudinal income change. To this end, changes in the income levels of VI and non-VI groups in a representative sample cohort dataset of the Korean National Health Insurance Service (KNHIS) were followed annually for 5 years. The groups’ initial income levels and demographic factors were matched, and for the sensitivity analyses, subgroup analyses according to age, sex, and severity of VI were performed.

## Results

The sociodemographic variables between the VI and matched control groups are summarized in Table 1. The standardized difference shows a 0.00–0.15 distribution, indicating a small approximate intergroup difference of under 10% non-overlapping among the total variables.

**Table 1:** General characteristics of participants at baseline.

Characteristics	Total participants		
	Visual impairment (n, %)	Control (n, %)	Standardized difference
**Age (years old)**			0.00
40–44	351 (7.4)	1404 (7.4)	
45–49	504 (10.6)	2016 (10.6)	
50–54	668 (14.0)	2672 (14.0)	
55–59	804 (16.9)	3216 (16.9)	
60–64	895 (18.8)	3580 (18.8)	
65–69	698 (14.7)	2792 (14.7)	
70–74	493 (10.4)	1972 (10.4)	
75–79	253 (5.3)	1012 (5.3)	
80–84	84 (1.8)	336 (1.8)	
85+	8 (0.2)	32 (0.2)	
**Sex**			0.00
Male	2917 (61.3)	11,668 (61.3)	
Female	1841 (38.7)	7364 (38.7)	
**Income (range categories)**			0.00
0 (lowest)	104 (2.2)	416 (2.2)	
1	490 (10.3)	1960 (10.3)	
2	377 (7.9)	1508 (7.9)	
3	411 (8.6)	1644 (8.6)	
4	448 (9.4)	1792 (9.4)	
5	412 (8.7)	1648 (8.7)	
6	474 (10.0)	1896 (10.0)	
7	396 (8.3)	1584 (8.3)	
8	447 (9.4)	1788 (9.4)	
9	569 (12.0)	2276 (12.0)	
10 (highest)	630 (13.2)	2520 (13.2)	
**Region of residence**			0.00
Urban	1908 (40.1)	7632 (40.1)	
Rural	2850 (59.9)	11,400 (59.9)	
**BMI groups**^**†**^			0.04
Underweight	141 (3.0)	621 (3.3)	
Normal	1637 (34.4)	6764 (35.5)	
Overweight	1314 (27.6)	5133 (27.0)	
Obese I	1524 (32.0)	6037 (31.7)	
Obese II	142 (3.0)	477 (2.5)	
**Smoking status**			0.02
Nonsmoker	3258 (68.5)	12,891 (67.7)	
Past smoker	459 (9.7)	1795 (9.4)	
Current smoker	1041 (21.9)	4346 (22.8)	
**Alcohol consumption**			0.05
Non-consumer	3498 (73.5)	13,527 (71.1)	
≥ 1 time a week	1260 (26.5)	5505 (28.9)	
**Systolic blood pressure**			0.03
< 120 mmHg	1132 (23.8)	4705 (24.7)	
120–139 mmHg	2125 (44.7)	8602 (45.2)	
≥ 140 mmHg	1501 (31.6)	5725 (30.1)	
**Diastolic blood pressure**			0.03
< 80 mmHg	1759 (37.0)	7173 (37.7)	
80–89 mmHg	1662 (34.9)	6856 (36.0)	
≥ 90 mmHg	1337 (28.1)	5003 (26.3)	
**Fasting blood glucose**			0.07
< 100 mg/dL	2899 (60.9)	12,199 (64.1)	
100–125 mg/dL	1306 (27.5)	4962 (26.1)	
≥ 126 mg/dL	553 (11.6)	1871 (9.8)	
**Total cholesterol**			0.02
< 200 mg/dL	2496 (52.5)	9855 (51.8)	
200–239 mg/dL	1566 (32.9)	6426 (33.8)	
≥ 240 mg/dL	696 (14.6)	2751 (14.4)	
**CCI score**			0.13
0	2708 (56.9)	11966 (62.9)	
1	887 (18.6)	2825 (14.8)	
2	502 (10.6)	1770 (9.3)	
3	260 (5.5)	889 (4.7)	
≥ 4	401 (8.4)	1582 (8.3)	

CCI, Charlson comorbidity index.

*Chi-square test. Significance at P < 0.05.

^†^BMI groups (BMI, body mass index, kg/m2) was categorized as < 18.5 (underweight), ≥ 18.5 to < 23 (normal), ≥ 23 to < 25 (overweight), ≥ 25 to < 30 (obese I), and ≥ 30 (obese II).

Table 2 shows the changes in the mean values of income level, beginning with the initial level and proceeding over the 5-year course of the annual follow-ups. The income levels increased according to the duration of follow-up in both the VI and non-VI groups. In the VI group, the income levels post 3–5 years were significantly increased relative to the initial value (P = 0.018, < 0.001 and < 0.001), while the income levels post 1 and 2 years were not (all P > 0.05). In the control group, the income levels were increased significantly each year from post 1 year through post 5 years (all Ps < 0.001). The rate of change in income between time and VI were significant (interaction effects, P = 0.003). The difference of mean value for income level between the VI and non-VI groups was estimated to be − 0.014 (P = 0.771) by the adjusted model.

**Table 2:** Distribution/repartition of mean of income value over time in visual impairment and non-visual impairment groups.

Characteristics	Paired t-test						Interaction^†^	Linear mixed model^¶^	
	Previous	Post 1 yr	Post 2 yr	Post 3 yr	Post 4 yr	Post 5 yr	P-value	EV^§^	P-value
**Total participants (n = 23,790)**									
Visual impairment (mean, P-value)	5.662	5.621 (0.108)	5.702 (0.241)	5.752 (0.018*)	5.803 (< 0.001*)	5.824 (< 0.001*)	0.003^‡^	− 0.014	0.771
Non-visual impairment (mean, P-value)	5.662	5.776 (< 0.001*)	5.853 (< 0.001*)	5.865 (< 0.001*)	5.946 (< 0.001*)	6.001 (< 0.001*)			
EV by time of F/U (P-value)		0.00082 (0.987)	0.00031 (0.995)	− 0.00045 (0.993)	− 0.00016 (0.997)	0.00051 (0.992)			
**Age < 60 years old, men (n = 7645)**									
Visual impairment (mean, P-value)	5.831	5.810 (0.625)	5.812 (0.711)	5.801 (0.576)	5.765 (0.304)	5.773 (0.379)	< 0.001^‡^	− 0.019	0.813
Non-visual impairment (mean, P-value)	5.831	6.006 (< 0.001*)	6.079 (< 0.001*)	6.044 (< 0.001*)	6.099 (< 0.001*)	6.106 (< 0.001*)			
EV by time of F/U (P-value)		0.00993 (0.904)	0.00918 (0.911)	0.008423 (0.918)	0.01036 (0.900)	0.00943 (0.909)			
**Age < 60 years old, women (n = 3990)**									
Visual impairment (mean, P-value)	5.188	5.046 (0.035*)	5.162 (0.693)	5.321 (0.135)	5.332 (0.141)	5.340 (0.149)	0.035^‡^	− 0.020	0.861
Non-visual impairment (mean, P-value)	5.188	5.389 (< 0.001*)	5.456 (< 0.001*)	5.515 (< 0.001*)	5.583 (< 0.001*)	5.661 (< 0.001*)			
EV by time of F/U (P-value)		0.03463 (0.765)	0.03033 (0.794)	0.03467 (0.765)	0.02813 (0.810)	0.02515 (0.830)			
**Age ≥ 60 years old, men (n = 6940)**									
Visual impairment (mean, P-value)	5.812	5.801 (0.822)	5.885 (0.248)	5.964 (0.034)	6.107 (< 0.001*)	6.192 (< 0.001*)	0.973	− 0.002	0.983
Non-visual impairment (mean, P-value)	5.812	5.845 (0.195)	5.934 (< 0.001*)	5.971 (< 0.001*)	6.071 (< 0.001*)	6.168 (< 0.001*)			
EV by time of F/U (P-value)		− 0.00930 (0.920)	− 0.00788 (0.932)	− 0.00795 (0.931)	− 0.00762 (0.933)	− 0.00615 (0.947)			
**Age ≥ 60 years old, women (n = 5215)**									
Visual impairment (mean, P-value)	5.579	5.543 (0.527)	5.713 (0.052)	5.735 (0.068)	5.827 (0.012*)	5.803 (0.034*)	0.401	− 0.005	0.965
Non-visual impairment (mean, P-value)	5.579	5.642 (0.021*)	5.717 (< 0.001*)	5.735 (< 0.001*)	5.844 (< 0.001*)	5.902 (< 0.001*)			
EV by time of F/U (P-value)		− 0.00352 (0.975)	− 0.00434 (0.969)	− 0.0061 (0.957)	− 0.00413 (0.971)	− 0.0024 (0.983)			

EV, Estimated value; CCI, Charlson Comorbidity Index.

*Paired t-test based on previous income, significance at P < 0.05.

^†^Interaction effects between time and group.

^‡^Interaction effects by linear mixed model, significance at P < 0.05.

^§^Estimated value of linear mixed model for visual impairment group based on control group.

^¶^Fixed effects were age, sex, region of residence, visual impairment, and time of measurement. Random effects were BMI, systolic blood pressure, diastolic blood pressure, fasting blood glucose, total cholesterol, smoking, alcohol consumption, and CCI scores.

In our subgroup analysis for age and sex, time and VI’s interaction effects impacting income level were significant for the < 60-years-old subgroups of both men and women (P < 0.001 and 0.035). The differences of mean value for income level between the two groups were estimated to be − 0.019 and − 0.020, respectively, in these subgroups (P = 0.813 and 0.861). In the subgroup of men aged < 60 years old, the income level of the VI group did not differ from the initial level to post 1 year through post 5 years; by contrast, it significantly increased from the initial level to post 1–5 years (all Ps < 0.001) in the non-VI group. Similarly, in the subgroup of women < 60 years old, the income level of the VI group decreased at post 1 year (P = 0.035) and did not differ thereafter, whereas the income level of the non-VI group increased each year (post 1–5 year, each P < 0.001). The ≥ 60-year-old men and ≥ 60-year-old women did not show any significant interaction effects between time and VI for income level.

According to the severity of VI, the longitudinal changes of income level were analyzed as shown in Table 3. In the mild-to-moderate VI group (n = 4170), the income levels of post 3–5 years were higher than the initial value (P = 0.028, < 0.001 and < 0.001, respectively), while the non-VI group showed increased income throughout the post 1–5 years (all Ps < 0.001). The rate of change in income between time and VI for income level was significant (P = 0.005), and the adjusted difference of mean value for income level between the VI and non-VI groups was estimated to be − 0.016 (P = 0.767) in this subgroup. Meanwhile, the severe VI group (n = 588) did not show any significant interaction between time and VI for income level (P = 0.229).

**Table 3:** Difference in mean values of income between initial value and post 5-year values of visual impairment in visual impairment and non- visual impairment groups by severity of disability.

Characteristics	Paired t-test						Interaction^†^	Linear mixed model^¶^	
	Previous	Post 1 yr	Post 2 yr	Post 3 yr	Post 4 yr	Post 5 yr	P-value	EV^§^	P-value
**Mild-to-moderate (n = 4170 for visual impairment, n = 16,680 for control)**									
Visual impairment (mean, P-value)	5.688	5.655 (0.220)	5.733 (0.226)	5.773 (0.028*)	5.823 (0.001*)	5.847 (< 0.001*)	0.005^‡^	− 0.016	0.767
Non-visual impairment (mean, P-value)	5.688	5.804 (< 0.001*)	5.883 (< 0.001*)	5.896 (< 0.001*)	5.976 (< 0.001*)	6.025 (< 0.001*)			
EV by time of F/U (P-value)		− 0.00184 (0.972)	0.00341 (0.990)	0.00263 (0.960)	− 0.00212 (0.968)	0.00355 (0.947)			
**Severe (n = 588 for visual impairment, n = 2352 for control)**									
Visual impairment (mean, P-value)	5.478	5.380 (0.197)	5.488 (0.889)	5.598 (0.388)	5.654 (0.442)	5.655 (0.609)	0.229	− 0.032	0.824
Non-visual impariment (mean, P-value)	5.478	5.598 (0.001*)	5.665 (< 0.001*)	5.664 (< 0.001*)	5.739 (< 0.001*)	5.821 (< 0.001*)			
EV by time of F/U (P-value)		0.00174 (0.990)	− 0.01275 (0.928)	− 0.00812 (0.954)	− 0.01147 (0.935)	− 0.00481 (0.973)			

EV, Estimated value; CCI, Charlson Comorbidity Index.

*Paired t-test based on previous income, significance at P < 0.05.

^†^Interaction effects between time and group.

^‡^Interaction effects by linear mixed model, significance at P < 0.05.

^§^Estimated value of linear mixed model for visual impairment group based on control group.

^¶^Fixed effects were age, sex, region of residence, visual impairment, and time of measurement. Random effects were BMI, systolic blood pressure, diastolic blood pressure, fasting blood glucose, total cholesterol, smoking, alcohol consumption, and CCI scores.

## Discussion

In this study, in order to investigate the impact of VI on longitudinal income change, health insurance data was analyzed for a large national population cohort. We performed an in-depth analysis of the effects of VI on income changes in both the VI and matched non-VI groups. The income level increased in both groups over the course of 5 years, but the increase was smaller in the VI group. Although we had matched the initial income level of the VI and non-VI groups, the income gap between them widened over time. The interaction of time and VI for income level was significant in a linear mixed model. A similar finding was observed in a subgroup analysis of < 60-year-old participants.

Although there have been a few studies on VI and income level to date^6,13,15,16^, the economic consequences of VI have rarely been evaluated in representative samples of national populations. Also, the present study’s longitudinal design contrasts with the majority of studies published thus far, which have been of cross-sectional design^3,4,6,14,17^. The results of cross-sectional studies typically have limited implications due to the lack of temporality of risk factor data; this means that the causal relationship between, for example, VI and income level, would have to be interpreted with caution. The present study, on the other hand, using the linear mixed model, was able to estimate the interaction of time and VI for income level.

Interestingly, our results showed that income level increased in both the VI group and the non-VI group. There are several possible explanations for this. First, VI participants could also think of a case wherein they had been in higher-paying occupations as their experience built up^18–20^. Next, currency inflation could have made the income figures of even VI participants appear to have improved macroscopically. The finding of income growth, in itself, in the VI group might be taken as an encouraging result. However, the rate of increase in income level was, characteristically, lower than that of the non-VI group. Moreover, most of the VI group may well have had a lower baseline income compared with the non-VI group; indeed, several cross-sectional studies have noted low baseline income for VI relative to non-VI individuals^5,21,22^. And although we included initial-income-matched non-VI participants, income growth was lower in the VI group. This is indicative of a wider gap in total income/assets between the VI and non-VI groups in the real world.

As could be expected, individuals with VI are known to have less economic capacity. Brezin et al.^13^ found monthly household incomes to be lower for the low vision (€1255) and blindness (€1587) groups than for those having no visual problems (€1851). In Britain, the risk of poor vision has been associated with social class (i.e., unskilled manual workers)^23^. People suffering VI have been deemed to be at greater risk of unemployment, permanent disability, being a member of the working class, lacking skills-development opportunity, being less recognized for their work, and earning an inadequate income^24^.

As other aspect of the consequences of VI, the increased expenditures in the VI group are a cause of income inequality. In the USA, VI groups have been significantly associated with higher medical care expenditures, a greater number of informal care days, and a decrease in health utility^5^. In addition, total non-medical costs associated with VI are considerable, and the preponderant economic consequences of visual impairment lie beyond healthcare systems^25^.

Aging by itself is a source of disability and a universal risk factor associated with VI. Rates of VI and blindness have been documented to increase sharply with age, beginning at about 65 to 70 years^22,26,27^. In the present study, the effect of age on income change was adjusted for by subdividing and analyzing age based on the age of 60. Even after this adjustment, under the age of 60, both men and women showed a greater increase of income in the non-VI group than in the VI group. Interestingly, for those *over* the age of 60, contrastingly, income change over time between the VI group and the non-VI group was *not* significant. The younger age group, certainly, would be expected to be economically more active than the older age group. Therefore, for them, the impact of VI on employment status, working performance and income level could be especially strong. More research is needed to assess whether such income differences as shown in our under-60 group between VI sufferers and non-VI individuals can be explained by other socioeconomic differences.

In present study, income changes also were analyzed according to severity of VI. In the mild-to-moderate group (n = 4170 for VI), most of the study subjects showed an interaction effect between time and VI for income level. However, the severe VI group (n = 588 for VI) showed no significant income-change differences over time. We considered that the relatively small size of the severe VI group was insufficient to secure the statistical power.

In addition to visual function, educational level and SES also interact with each other^2–7,12^. VI, educational level, and SES act in similar though different ways to produce low income levels. That is, the effect of an individual’s VI on his/her income change might not be direct only, but might also emerge from other, intermediary and perhaps complex determinants that remain, pending investigation.

There are some limitations to this study. First, limitations of available data precluded us from considering the leading causes of VI. Due to the study design’s use of KNHIS data, there was no specific data on VI causes. In epidemiological investigations, the major cause of VI has been mostly age-related macular degeneration in developed countries, and cataract in under-developed countries^28–31^. Analysis of the causes of VI could help to understand economic inequality caused by VI. Second, selection bias may have influenced our results. The use of registers to estimate VI prevalence is in any case controversial, since a high proportion of subjects thus impaired do not register^32,33^. Moreover, in Korea, application for registration of disability is directly made by the individual him/herself; and there is a strong possibility that a high proportion of VI persons who do register are those who experience economic difficulty. Third, since EV values were calculated with the covariate-adjusted linear mixed model, some of them were positive, indicating that income in the VI was higher than in the non-VI group. But because the absolute value of EV was nearly 0, a minimal change of value might affect the (+)/(−) status. Forth, the true date of onset of vision impairment is unknown; all that is known is the date of registration as visually impaired. Thus, caution is required in interpreting causality between VI and income level. Finally, the criteria used in the current study to define VI was based on the Korean government’s visual disability standard. According to the WHO and ICD 11 definition, a person is said to be visually impaired if the presenting visual acuity in the better eye is worse than 6/12 (< 20/40). Therefore, it is possible that severe VI was mainly included in this study, due to the rather strict VI criteria of the Korean government. Additionally, since the legal VI registration criteria may differ by country, any generalization of the present results should be done with caution.

Despite these limitations, this study reports, based on a longitudinal database, nationwide estimates of how VI affects income change according to subject age and severity of VI. We showed that in a large representative sample of Korea, the growth in the income level of the VI group was less than that of a non-VI group matched for age, sex, region of residence and income level. Although additional research is needed to more thoroughly elucidate and target the drivers of disparity, our findings identify areas requiring improvement for people with VI. Based on these data, a specific nationwide database for the SES of VI could be compiled, and in turn, policies would be formulated to provide the most appropriate financial and social assistance.

## Methods

This study was authorized by the Ethics Committee of Hallym University (2019-10-023). Written informed consent was waived by the Institutional Review Boards of Hallym University Sacred Heart Hospital. In all of the analyses, the Ethics Committee guidelines and regulations were strictly adhered to. In Korea, a representative sample cohort database comprising approximately one million people has been provided by the KNHIS. The database includes medical care histories listed by diagnostic/treatment codes, socioeconomic data, life and death information, and individual disability over a period ranging from 2002 to 2015^34–36^. Many previous studies in Korea have used the KNHIS premiums as an indicator of income^37–39^.

### Definition of visual impairment

Participants found to have VI where those who had registered as VI persons at the Ministry of Health and Welfare. We excluded those with co-disabilities. In Korea, legal VI is defined as the presence of any of the following 4 conditions that show stabilization after at least 6 months of treatment and are not reversed by medication or surgery: (1) best-corrected visual acuity (BCVA) ≤ 20/1000 in the worse eye, (2) BCVA ≤ 20/100 in the better eye, (3) a binocular visual field < 1/2, and (4) visual field ≤ 10° from the visual axis for both eyes. The patient must submit a medical certificate issued by an ophthalmologist regarding the BCVA, visual field, and the possible reason for VI before being registering as visually impaired. With properly documented evidence of a VI, an assessment committee discusses the feasibility of the VI registration. In Korea, the degree of VI is typically divided into 6 grades according to the its severity; in the database, the data are then divided into two grades (mild-to-moderate VI group, grades III-VI; severe VI group, grades I-II).

### Definition of income

Income level was divided into deciles of population based on KNHIS annual premiums (Supplementary Table 1). The unit of the income values reported is US dollars. Medical Aid beneficiaries were added to the lowest income level^40^. Income change for participants was defined as an income-level change between the income prior and closest to the day of VI registration and the income 5 years after that.

### Participant selection

**Figure 1:**
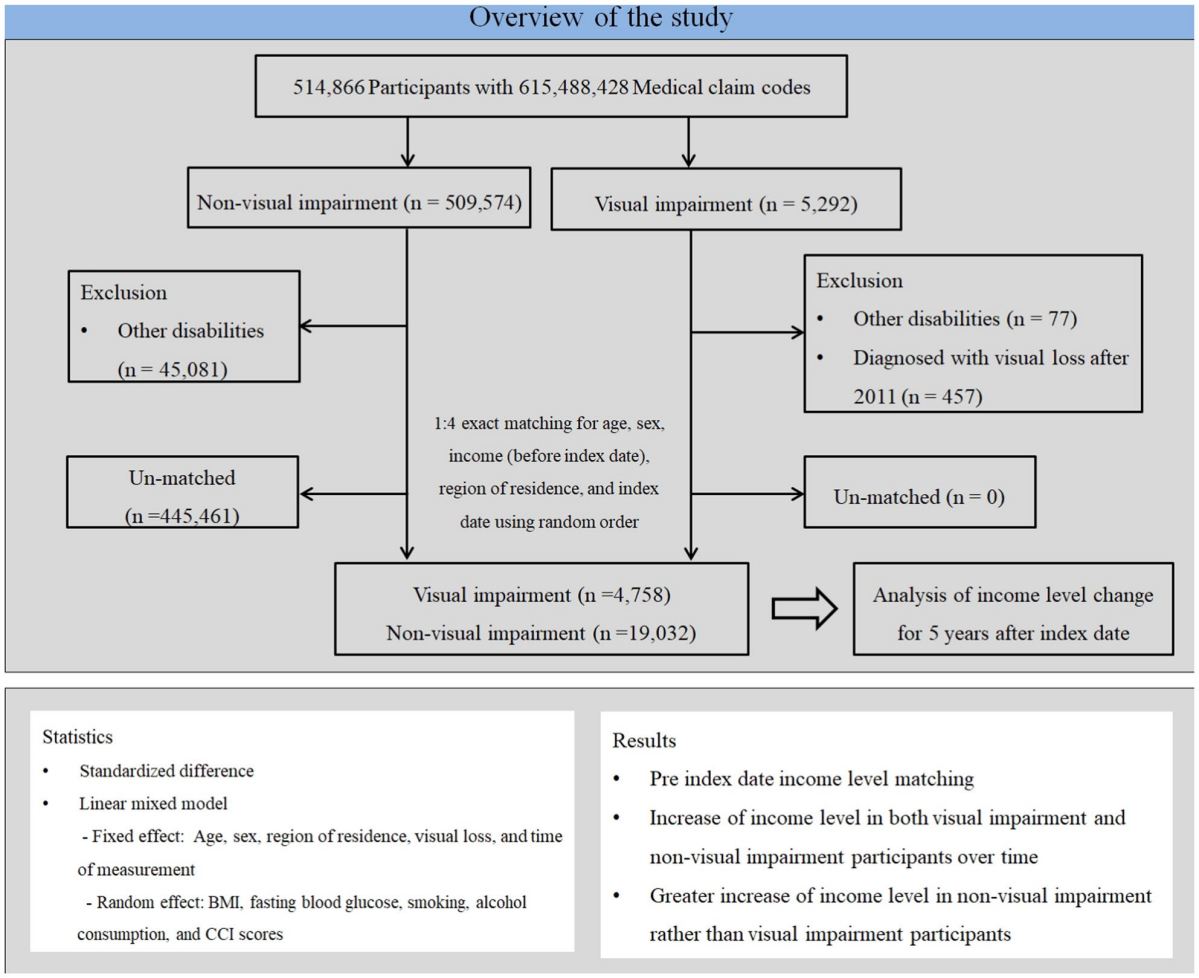
Schematic illustration of participant selection process used in present study. Among a total of 514,866 participants, 4758 visual impairment (VI) participants were 1:4 matched with 19,032 non-VI participants for age, sex, income level, and region of residence.

The VI group was selected from 514,866 participants for whom 615,488,428 medical claim codes had been registered between the years 2002 and 2015 (n = 5292). The control group was compiled of participants not defined as VI during the years 2002 through 2015 (n = 509,574). VI participants diagnosed with other disabilities (n = 77) or with VI after 2011 (n = 457) were excluded. VI participants were matched with participants (non-VI group) who had never been diagnosed with a VI or other disabilities from 2002 through 2015. Non-VI participants were excluded if they had been diagnosed with disabilities (n = 45,081). VI participants were 1:4 matched with non-VI participants for age, sex, initial income on index date, and region of residence. The participants serving as the control were randomly selected in order to minimize selection bias. The index date of each VI participant was set as the time of diagnosis of VI. The index date of each non-VI participant was set as the index date of the matched VI participant. Therefore, each VI/ non-VI participant matched pair had the same index date. During the matching procedure, 445,461 non-VI participants were excluded. Ultimately, 4758 VI participants were 1:4 matched with 19,032 non-VI participants (Fig. 1). The information on lost persons or deaths is summarized in Supplementary Table 2.

### Covariates

All covariates were collected at baseline. The following ten age groups were formed: 40–44, 45–49, 50–54…, 80–84, and 85 + years old. Residence regions were grouped into urban (Seoul, Busan, Daegu, Incheon, Daejeon, Gwangju, Ulsan) and rural (Gangwon, Gyeonggi, Chungcheongbuk, Chungcheongnam, Gyeongsangbuk, Gyeongsangnam, Jeollabuk, Jeollanam, Jeju) areas.

Tobacco smoking was classified based on the current smoking status (current smoker, past smoker, and nonsmoker) of participants. Alcohol consumption was categorized based on its frequency (≥ 1 time a week and non-consumer). Obesity was determined according to body-mass index (BMI, kg/m^2^), BMI having been categorized as follows: < 18.5 (underweight), ≥ 18.5 to < 23 (normal), ≥ 23 to < 25 (overweight), ≥ 25 to < 30 (obese I), and ≥ 30 (obese II), based on the Asia–Pacific criteria of the Western Pacific Regional Office (WPRO) 2000^41^. Also investigated were systolic blood pressure (SBP) and diastolic blood pressure (DBP) along with fasting blood glucose and total cholesterol. Missing BMI (23/23,790 [0.097%]), SBP (17/23,790 [0.071%]), DBP (17/23,790 [0.071%]), fasting blood glucose (31/23,790 [0.130%]), and total cholesterol (48/23,790 [0.202%]) values were replaced by the mean values of each variable for the final selected participants.

The Charlson Comorbidity Index (CCI) has been used widely to measure disease burden according to 17 comorbidities. The CCI score is reflective of disease severity and number both, and is recorded as a continuous variable (range: 0 [no comorbidities]–29 [multiple comorbidities])^42,43^.

### Statistical analyses

For the sensitivity analyses, the baseline values of SBP, DBP, fasting blood glucose, and total cholesterol were classified and compared between the VI and non-VI groups. The differences in the means and prevalence of the baseline characteristics were compared using standardized differences. An SD of 0.2 means approximately 14.8% non-overlapping (a more equal overlap means no difference between the case–control groups). When the SD value is 0.2, the difference between the two groups is small; when the SD value is 0.5, the difference is medium (33% does not overlap), and when the SD value is 0.8, about 50% does not overlap, which means that the case–control group is quite different^44^. The differences in the mean values of income between the initial index date and the 5-years-post-VI-registration date were compared using the paired t-test.

The decile partition of income distribution was treated as a categorical variable. To estimate the interaction and estimated value (EV) of repeated measures data, a covariate-adjusted linear mixed model with was used. Age, sex, region of residence, VI, and time of measurement were used as the independent variables and fixed effects. BMI, SBP, DBP, fasting blood glucose, total cholesterol, smoking, alcohol consumption, and CCI scores were used as random effects. A first-order autoregressive model was selected as the repeated covariance type, considering the correlation of each participant's iteration. The statistical analysis of the linear mixed model proceeds as follows:

$$ Yij \, = \, \beta 0 \, + \, \beta 1Xi \, + \, \beta pZip \, + \, \beta 3Xi*Tij \, + \, \beta Tij \, + \, g0i \, + \, Ui \, + \, \varepsilon ij; $$

where Yij is value of income of participant i at assessment moment j; X is the explanatory variable (VI, non-VI); Z is the matrix of covariates; Tij is the time between assessment j and the baseline of participant i; g0 is the random effect for the intercept; U is the random effect for the estimate, and p is the count of covariates.

Additionally, subgroup analyses according to age, sex, and severity of VI were performed to identify either consistency of or large differences in the magnitude of income change among the different categories. In the subgroup analyses, we subdivided the participants according to age and sex (< 60 years old and ≥ 60 years old; men and women). According to severity, VI was divided into mild-to-moderate VI and severe VI groups.

We performed two-sided analyses and determined statistical significance based on P values < 0.05. SAS version 9.4 (SAS Institute Inc., Cary, NC, USA) was used in the analyses.

## Supplementary Information

Supplementary Table 1.

Supplementary Table 2.

## Data Availability

Data supporting the findings of the current study are available from the corresponding author on reasonable request.
