# Supplementary material for: A longitudinal study of the association between visual impairment and income change using a national health screening cohort
Source: Sci Rep. 2022 Jan 19;12:958. doi: 10.1038/s41598-022-05003-6 (PMC8770619; doi:10.1038/s41598-022-05003-6)
Supplement: Supplementary file 1 — Supplementary Table 1. [file 41598_2022_5003_MOESM1_ESM.docx]

**Supplementary Table 1** Status of income levels by year

| Income  level | 2002 | 2003 | 2004 | 2005 | 2006 | 2007 | 2008 | 2009 | 2010 | 2011 | 2012 | 2013 | 2014 | 2015 |
| --- | --- | --- | --- | --- | --- | --- | --- | --- | --- | --- | --- | --- | --- | --- |
| Total  n, (%) | 514,866  (100) | 514,515  (100) | 512,802  (100) | 509,900  (100) | 506,189  (100) | 503,007  (100) | 498,566  (100) | 494,192  (100) | 490,255  (100) | 487,835  (100) | 483,421  (100) | 478,740  (100) | 472,214  (100) | 467,132  100 |
| 0  n, (%) | 515  (0.10) | 1,785  (0.35) | 3,662  (0.71) | 6,123  (1.20) | 7,482  (1.48) | 8,529  (1.70) | 8,764  (1.76) | 8,061  (1.63) | 8,415  (1.72) | 8,550  (1.75) | 8,324  (1.72) | 8,341  (1.74) | 8,345  (1.77) | 9,488  (2.03) |
| 1  n, (%) | 45,828  (8.90) | 43,806  (8.51) | 40,699  (7.94) | 39,620  (7.77) | 37,069  (7.32) | 37,002  (7.36) | 37,227  (7.47) | 36,751  (7.44) | 37,019  (7.55) | 37,069  (7.60) | 37,180  (7.69) | 37,192  (7.77) | 37,630  (7.97) | 37,530  (8.03) |
| 2  n, (%) | 35,642  (6.92) | 35,330  (6.87) | 35,242  (6.87) | 32,652  (6.40) | 31,046  (6.13) | 30,875  (6.14) | 29,679  (5.95) | 28,941  (5.86) | 31,404  (6.41) | 32,078  (6.58) | 31,179  (6.45) | 29,593  (6.18) | 29,213  (6.19) | 28,126  (6.02) |
| 3  n, (%) | 36,580  (7.10) | 33,516  (6.51) | 34,395  (6.71) | 35,732  (7.01) | 34,254  (6.77) | 32,836  (6.53) | 33,351  (6.69) | 32,252  (6.53) | 31,050  (6.33) | 31,992  (6.56) | 30,307  (6.27) | 31,576  (6.60) | 31,161  (6.60) | 30,514  (6.53) |
| 4  n, (%) | 37,489  (7.28) | 36,704  (7.13) | 35,562  (6.93) | 38,012  (7.45) | 36,842  (7.28) | 35,428  (7.04) | 33,722  (6.76) | 33,340  (6.75) | 32,670  (6.66) | 32,099  (6.58) | 31,113  (6.44) | 30,444  (6.36) | 30,904  (6.54) | 31,779  (6.80) |
| 5  n, (%) | 39,124  (7.60) | 39,609  (7.70) | 38,204  (7.45) | 37,563  (7.37) | 37,365  (7.38) | 38,393  (7.63) | 37,646  (7.55) | 37,159  (7.52) | 35,881  (7.32) | 36,210  (7.42) | 35,016  (7.24) | 34,530  (7.21) | 34,381  (7.28) | 33,902  (7.26) |
| 6  n, (%) | 42,577  (8.27) | 41,608  (8.09) | 41,939  (8.18) | 42,082  (8.25) | 44,304  (8.75) | 42,851  (8.52) | 41,430  (8.31) | 41,649  (8.43) | 40,851  (8.33) | 40,103  (8.22) | 39,449  (8.16) | 39,134  (8.17) | 39,333  (8.33) | 37,773  (8.09) |
| 7  n, (%) | 48,569  (9.43) | 49,693  (9.66) | 49,673  (9.69) | 48,491  (9.51) | 48,105  (9.50) | 48,214  (9.59) | 47,644  (9.56) | 46,929  (9.50) | 46,531  (9.49) | 46,199  (9.47) | 44,815  (9.27) | 44,702  (9.34) | 44,125  (9.34) | 44,171  (9.46) |
| 8  n, (%) | 57,208  (11.11) | 58,632  (11.40) | 57,914  (11.29) | 56,963  (11.17) | 56,251  (11.11) | 56,022  (11.14) | 56,328  (11.30) | 56,291  (11.39) | 55,707  (11.36) | 55,050  (11.28) | 54,429  (11.26) | 53,776  (11.23) | 53,822  (11.40) | 53,522  (11.46) |
| 9  n, (%) | 79,460  (15.43) | 79,648  (15.48) | 80,043  (15.61) | 79,199  (15.53) | 78,116  (15.43) | 76,621  (15.23) | 76,858  (15.42) | 76,214  (15.42) | 75,312  (15.36) | 74,416  (15.25) | 74,849  (15.48) | 73,630  (15.38) | 70,658  (14.96) | 68,777  (14.72) |
| 10  n, (%) | 91,874  (17.84) | 94,184  (18.31) | 95,469  (18.62) | 93,463  (18.33) | 95,355  (18.84) | 96,236  (19.13) | 95,917  (19.24) | 96,605  (19.55) | 95,415  (19.46) | 94,069  (19.28) | 96,760  (20.02) | 95,822  (20.02) | 92,642  (19.62) | 91,550  (19.60) |

Currency, converted to US dollar
