# Supplementary material for: A longitudinal study of the association between visual impairment and income change using a national health screening cohort
Source: Sci Rep. 2022 Jan 19;12:958. doi: 10.1038/s41598-022-05003-6 (PMC8770619; doi:10.1038/s41598-022-05003-6)
Supplement: Supplementary file 2 — Supplementary Table 2. [file 41598_2022_5003_MOESM2_ESM.docx]

**Supplementary Table 2** Dead participants by year after index date

|  | Dead participants | |
| --- | --- | --- |
|  | Visual impairment (n, %) | Non-visual impairment (n, %) |
| Post 1yr | 1 (1.0%) | 92 (11.6%) |
| Post 2yr | 11 (11.0%) | 179 (22.7%) |
| Post 3yr | 31 (31.0%) | 219 (27.7%) |
| Post 4yr | 27 (27.0%) | 235 (29.7%) |
| Post 5yr | 30 (30.0%) | 245 (31.0%) |
| Total dead participants during the entire follow-up period | 100 (100.0%) | 970 (100.0%) |
